# The ‘insanity’ of Lady Durham

**DOI:** 10.1177/0957154X211064952

**Published:** 2022-05-19

**Authors:** Ruth Paley

**Affiliations:** Visiting Fellow, Oxford Brookes University, UK

**Keywords:** Autism, Autism Spectrum Disorder, schizoaffective disorder, schizophrenia, Ticehurst Hospital, 19th century

## Abstract

This essay draws on evidence in a late nineteenth-century court case and surviving medical notes to provide a case study of a hitherto unidentified case of Autism Spectrum Disorder. The case is particularly interesting in that it not only appears to be the first identification of historical ASD in a female, but also because the patient subsequently developed symptoms of psychosis suggestive of schizophrenia or schizoaffective disorder. The unusual survival of detailed medical notes also throws light on the ways in which a difficult patient was treated by supposedly enlightened pioneers of psychiatry.

## Introduction

In the spring of 1885, the public were both entranced and appalled by revelations in the case of *Durham v. Durham*. John George Lambton, 3rd Earl of Durham, was trying to end his marriage to Ethel Elizabeth Louise Milner. The couple had married in October 1882 after something of a whirlwind courtship. Less than two years later the new Lady Durham’s behaviour had become so bizarre that she was declared to be insane – incurably so, according to her doctors. Durham was in no position to seek a divorce as the marriage had been consummated and there was no question of his wife’s adultery, which would have provided him with grounds for dissolving the marriage. The one option open to Durham if he wished to extricate himself was an annulment, but for that he needed to establish that she had been insane at the time of their marriage and therefore unable to give informed consent.

In choosing this option, he ensured the opposition of the Milner family who faced the possibility of being accused of deliberately and wilfully tricking Durham into marriage and who regarded the action as an attempt to disgrace them (HALS, DE/X317/F17, Edward Milner to his sister Mary Gerard, 25 Mar. 1885). They insisted that she had been sane at the time of the marriage, though the evidence certainly raises a suspicion that, even if Ethel Milner’s younger siblings saw nothing amiss, her mother and older sister, Mary Gerard, did realize that there was something odd about her behaviour. Before the ceremony, Mrs Milner asked Durham to send his bride a letter reassuring her that she had nothing to fear on their wedding night and that she should ‘be plucky and keep up her courage’.^1^ She committed suicide before the trial, convinced that if she testified she would be found guilty of perjury.^2^ Some of Mary Gerard’s explanations of her sister’s behaviour are more suggestive of a determination to invent plausible excuses than to clarify events.

The ensuing hearing lasted nine days and is reputed to have cost Durham over £20,000.^3^ Dubbed by one newspaper as the ‘Hannen Show’ after the name of the presiding judge, an unusually crowded courtroom heard from a parade of witnesses who either testified to Lady Durham’s peculiarities both before and during her marriage or who described her as an exceptionally fragile and shy young woman whose mental condition deteriorated only after her marriage. Durham lost his case when the judge handed down his verdict. That verdict has since become a landmark ruling on what constitutes the capacity to consent to marriage. In arriving at his conclusion, Hannen managed to reconcile the apparent contradictions in the evidence that had been presented to him. He created a coherent narrative in which an ultra-sensitive Ethel Milner had been pressurized by her ambitious older sister to marry Durham, despite being in love with Lord Burghersh, whose suit had been rejected by her family.^4^ Her guilt played on her mind, and Durham’s lack of consideration and ‘manly tenderness’ after their marriage completed its destruction.^5^ At least one newspaper declared that the case stood as a warning to men ‘who marry for the sake of a pretty face, and without much regard to the disposition or intellectual faculties of the owner’.^6^ Another reporter took the opportunity to pontificate about the evils of ‘match-making mothers and match-making sisters’.^7^ Yet another noted that Durham’s legal gamble had ruined his reputation and wrecked the political career on which he had embarked.^8^ That his reputation was in tatters was confirmed when he was blackballed by the prestigious Marlborough Club just one day after the verdict was delivered (HALS, DE/X317/F12, E. Reilly to Mary Gerard, 11 Mar. 1885).^9^

## What was wrong with Lady Durham?

The evidence suggests that what Lady Durham’s friends, relatives and medical attendants found inexplicable about her behaviour – and the reason for the marked deterioration that occurred after her marriage – is likely to be understood by a modern clinician as suggestive of Autism Spectrum Disorder (ASD). Individuals with ASD exhibit a very wide range of characteristics which, broadly speaking, can be grouped into three main clusters: difficulties with social interactions, problems with communication and language, and a lack of imagination or rigidity of thinking. As will be seen, Lady Durham’s behaviour fitted all three of those clusters. Of course, a diagnosis of ASD was not available to doctors in the nineteenth century. Autism in children was first described by Leo Kanner in 1943, but it was initially regarded as a form of childhood schizophrenia. Widespread recognition of the condition as a developmental disorder did not emerge until the second half of the twentieth century. By the second decade of the twenty-first century, it was estimated that in developed countries the disorder affected at least 1.5 per cent of children (Lyall et al., 2017: 82).

The emergence of ASD as an apparently new phenomenon and the difficulties in securing adequate recognition of the condition spurred attempts to confirm the legitimacy of the diagnosis by establishing that it had always existed but had gone unrecognized. The survival of evidence that would enable us to locate autistic individuals in the past is obviously problematic. Nevertheless, some tentative identifications have been attempted; they include some of the ‘Holy Fools’ of Russia (Challis and Dewey, 1974), the feral child known as the ‘Wild Boy of Aveyron’ (Lane, 1977) and Hugh Blair of Borgue (Houston and Frith, 2000). All are male, reflecting something of the gender bias that infuses both archival survivals in general and the incidence of modern diagnoses of autism. Kanner’s (1943) original study on a small group of children identified four times as many boys as girls. Hans Asperger, writing in 1944, thought the condition did not affect girls at all.^10^ Estimates of the ratio of autistic males to females ranges from 2:1 to 16:1, with explanations for the disparity variously rooted in genetics or cultural assumptions about gender or a mixture of both (Skuse, 2000; Wing, 1981, 1993). The true level of ASD in females remains impossible to assess, since a significant proportion of autistic females are likely to be misdiagnosed or not diagnosed at all (Hull et al., 2017; Schuck, Flores and Fung, 2019). As a result, there is comparatively little research focusing specifically on women. Whether or not Lady Durham was suffering from ASD, her plight throws a light on the mechanisms that enable mental health issues in females to be masked, revealing as it does the ways in which explanations of her behaviour were filtered by the gendered assumptions of observers. A century and a half after Lady Durham was thought to have been driven mad by her brutish husband, gender stereotypes still enable a remarkable amount of leeway when assessing the behaviour of females. In Lady Durham’s case, the survival of medical case notes covering nearly four years of her confinement in a private asylum provides additional levels of complexity, revealing as they do the subsequent discovery that Lady Durham not only suffered from delusions but also from auditory hallucinations. If Lady Durham was indeed autistic, then she also suffered from co-morbidity – a topic that has only attracted the interest of researchers in the recent past (Larson et al., 2017; Simonoff et al., 2008).

As in the case of Hugh Blair (Houston and Frith, 2000), the legal proceedings in *Durham v. Durham* produced extensive information about Lady Durham’s mental health. Those testifying included relatives, friends and servants, as well as prominent society doctors. Their testimony does not appear in the formal record and has to be pieced together from newspaper reports.^11^ The spring of 1885 saw a political crisis – over war in the Sudan and the death of General Gordon – which would drive Gladstone from office in the summer. There were also ongoing problems from Irish nationalists and a devastating accidental explosion at the Shoeburyness artillery site. Newspapers nevertheless made plenty of room for coverage of the Durham trial, variously billing it as ‘The extraordinary divorce case’, ‘Sensational divorce case’, ‘A countess in the divorce court’, or more pointedly ‘An unhappy marriage for beauty and position’.^12^ The development of a commercial telegraph network meant that reporters could circulate accounts to the regional press very quickly, so coverage was not restricted to the London papers. As the focus of reports varied somewhat, it is sometimes necessary to read several accounts in order to get a full picture. Those accounts included lengthy quasi-verbatim transcripts of witness testimony.^13^ Taken together with the detailed case notes, these sources enable us to examine how her mental condition could remain hidden until her marriage stripped away the security of Ethel Milner’s protective family environment.

## Courtship and marriage

Durham’s courtship of Ethel Milner began in June 1882 when they met at a house party for Ascot week. Durham was then 27 years old and she was five years younger. He had inherited his earldom, and an income of over £70,000 a year, three years earlier; he had also inherited his family’s political allegiances. The Lambtons had long been prominent Whigs and, along with their fellow Whigs, had shifted their allegiance to the Liberal party. Durham had previously lived openly with a mistress, but by 1882 that relationship had ended. He was clearly intent on entering public life – the Liberals picked him to move the address to the Queen at the opening of the parliamentary session in February 1883 – and for that he needed a suitable wife.^14^ Ethel Milner appeared to be a perfect choice, as she was not only exceptionally beautiful but came from an impeccable upper middle-class family. Her relatives included senior clergymen and military officers as well as members of the House of Commons. She was to be seen at major social events, always in the company of her sister Mary, who was six years older, and whose husband, William Gerard, would succeed his father as Baron Gerard in 1887.

Ethel Milner was the third of six surviving children of Henry Beilby Milner and his wife Charlotte (née Beresford). According to Mary Gerard, she had been lively as a little child, ‘a mischievous little kitten’, was fond of animals and gardening, and able to play games such as croquet, cricket and hare-and-hounds with her brothers. She had become ‘graver’ at about six or seven years of age when she went into the schoolroom – like most girls of her class, she was educated at home by a governess. As a young teenager she had acted in private theatricals. She had the accomplishments to be expected of an upper-class young woman; she could play the piano and had learned both French and German. Significantly her ability to cope with social interactions varied according to circumstance. She was shy in the presence of strangers but not with her family. She attended balls with her sister and danced at them; she had also assisted at a charity bazaar and went to the races, but ‘Upon all these occasions she was very shy and sensitive of what was said of her’.^15^ Her surviving letters are trite and formulaic, but they are written in a clear fluent hand.^16^

Her maternal grandfather, the archbishop of Armagh, stated that she was ‘the most sensible’ of his granddaughters, effectively appealing to the conventional gender stereotype when he further explained that she had ‘more gravity and less levity, was reserved in her manner and acted prudently’ as well as being sensitive and thoughtful. One could wish for further elucidation of the statement by Mary Gerard that ‘She wished to have her own way’ and resented ‘interference’ by her mother, but sadly no further questions or answers are recorded.^17^ Other witnesses on behalf of the Milners made it clear that she was able to participate in conversations in very small groups, but that she became silent and withdrawn if more than two or three people were present. As a result, she had the reputation of being rather stupid, something of which she was aware and resentful.^18^ Problems with social interactions were highlighted by contrasting descriptions of the way in which she reacted to her wedding presents. Members of her intimate social circle described her as engaged and interested in who had given what; but when her new acquaintances among Durham’s friends and relatives visited they found that, on the contrary, she showed no interest in them, seemed not to know who had given what, and remained silent and unresponsive.^19^

Durham’s account of their courtship, and her limited, monosyllabic conversations, makes it clear that she was unable to relax in his company.^20^ Her invariable answer to any question was ‘I don’t know’. As Durham’s attentions became more pointed and it was clear that a proposal was imminent, she set her dress on fire with a match and was badly burned. She paid little attention to the pain, and refused medical attention, being ‘very plucky’, according to her sister, but it was clearly a serious burn since it left a scar some two inches long. Durham’s witnesses intimated that she was unnaturally indifferent to pain and implied that this was a suicide attempt.^21^ They had good reason for doing so. Suicide was associated with insanity; furthermore, Lady Durham’s mother had hanged herself shortly before the trial, suggesting that her insanity might be hereditary.^22^ Mary Gerard insisted the burn was accidental, but descriptions of the incident make it rather more probable that it was deliberate self-harm, and Lady Durham appears to have admitted as much after being declared insane.^23^

Durham was sufficiently concerned by Ethel Milner’s taciturnity and lack of responsiveness that he asked Mary Gerard about it. She insisted that Ethel’s shyness with him was because she was ‘so very much in love with him’. She herself, she said, had also been shy before her marriage, a statement that reassured him, not only because it flattered his ego but because reactions to her testimony suggest that ‘shy’ and ‘taciturn’ were not words that could be applied to Mary Gerard any longer. Durham proposed the following day. He also told Ethel about his previous cohabitation, but she did not appear to understand the full import of the confession, merely asking if he had been engaged or married to his lover. When he sealed the engagement with a kiss, she did not respond but insisted that she had done something ‘dreadful’ without being able to specify just what that was.^24^ Her conduct became even more distant after the engagement – so much so that he asked her whether she had been forced to accept him and whether she wished to break the engagement. She replied ‘I don’t know’ to both questions. He noted her insistence on using notepaper from a particular desk (even though identical notepaper was available elsewhere) and that she would write and rewrite her letters several times even though they needed no improvement. She continued to be obsessive about notepaper and rewriting her letters after her marriage.^25^

Durham’s friends, relatives and servants all testified that when they were introduced to Ethel after the engagement, she was awkward and unable or unwilling to converse with them. She would stand until told to sit, but having sat it was difficult to persuade her to stand up again.^26^ Several witnesses referred to her reluctance to eat, toying with her food and eating very little.^27^ Her repetitive behaviours included a habit of only sitting on the edge of a chair, fidgeting with her hands or pulling her gloves on and off. During two long train journeys with Durham and others whom she knew only slightly, she either remained silent or muttered to herself. In an attempt to stir her into conversation, Durham joked that he would kiss their fellow passenger, Lady Lonsdale, but she was unable to understand the joking intent and interpreted it literally. On another occasion, Durham tried to get her to talk by saying that he would throw his hat over a hedge. Again she took the remark literally, replying ‘Oh please don’t. How dreadful.’^28^ When the couple left the church after their wedding, the new Lady Durham sat as far apart from her husband as possible; when they arrived at her mother’s house she could not, as convention demanded, get out of the carriage first and had to be almost pushed out. Then, despite the rain, she simply stood on the pavement until Durham gathered her train and took her indoors to the reception where she stood, according to her sister, shyly, or according Durham’s witnesses, looking dazed. Far from coming out of her shell, she continued to shrink from Durham and avoided his company.^29^ Three days after their wedding, Durham was so upset and angry about her behaviour that he threatened to send her home to her mother.^30^

Marriage to a wealthy aristocrat and budding politician inevitably increased the complexity of the social demands imposed on her. Her anxiety levels were so high that they prevented her from initiating the sort of interactions that convention demanded of someone in her social position. When Durham first took her to his country home, Lambton Castle, near Chester-le-Street, the tenantry turned out *en masse* to greet her, but she could not respond to them, and ‘kept her eyes fixed on the ground’. During the welcome, there was an accident, and a child was killed. Durham took his new wife to console the bereaved parents, but she stayed in the carriage and could not bring herself to shake hands with them. As hostess of dinner parties, it was her role to indicate the end of the meal by rising from the table, but she did not do so, more than once leaving her guests waiting for the signal to move for some 20 minutes until Durham himself stood up. She refused to interact with the servants in any way, one remarking that ‘She hung her head down’ and another that ‘She always looked down; never looked up’. She had knitting in her hands, but did nothing with it. She later told her sister that she was frightened that the servants would ignore her orders, though it is clear that she made no attempt to give any orders. She was similarly unable to deal with the local shopkeepers who were called in to take instructions for changes to the décor and soft furnishings of the sitting room that had been allocated to her. Taken to meet county society at a ball, she simply sat near the wall with her eyes fixed on the ground. Despite having a substantial wardrobe, she would wear only two of her dresses and her choice was not necessarily appropriate to the weather. She also developed at least two coping mechanisms: playing the piano alone and taking long walks, also alone.^31^

In February 1883, Durham’s aunt took Lady Durham to the House of Lords to hear Durham move the address to the Queen in February 1883 – a major event in his nascent political career. Later, she insisted that she had not been there and so had no recollection of hearing it. Durham was now so perturbed by his wife’s behaviour that he called in the distinguished society doctor Sir William Gull. During the consultation her responses to Gull’s questions were monosyllabic, and he concluded that she was ‘depressed, languid and her digestion was disordered’ – in other words that she was constipated. The *Daily News* account of some of the questions put to Gull during the trial imply that he told Durham that his wife should be treated like a child and ‘ought not to be worried with any brain work’, though Gull, a long-term family friend of the Milners, insisted that he did not remember making any such statement.^32^

Having already been subjected to constant change (taken to visit Durham’s friends and relations during the engagement, then on honeymoon, followed by a stay at Lambton Castle before returning to Durham’s London residence), Lady Durham’s behaviour deteriorated markedly when it was decided that it would be helpful for her health if the couple were to take a holiday in the south of France. They were accompanied by Lady Durham’s maid. During the trip, she took a sudden dislike to the maid, who had been in satisfactory service with the Milners for 10 years, and insisted she must be sacked.^33^ On their return, even her attitude to her family had changed, and her younger sister, Emily, noted that she had become ‘cold’. Her condition became public when she attended a ball in June 1883, and sat laughing, refusing to speak, dance or go into supper. On the assumption that his wife’s condition was caused by physical disorder, the following month Durham arranged for her to be seen by the eminent gynaecologists, James Matthews Duncan and William Playfair. It was Playfair who, concerned about her mental health, recommended that the pioneering psychiatrist, George Fielding Blandford, be called in.^34^

A second European trip did nothing to improve Lady Durham’s health; indeed she seems to have blanked the entire experience. On her return in September 1884, she was confined in The Knoll, a large private house with extensive grounds on Kingston Hill, where she was treated by Blandford and another pioneer psychiatrist, Henry Maudsley. Her attendants described her as childish, silent and tittering. When she was asked if she wished to return to Durham, she simply replied ‘I don’t know’. She repeatedly told her sister that she had done something dreadful, but as with her conversation with Durham, could not indicate what something dreadful actually meant. In December she attacked and bit one of the nurses, leading Blandford and Maudsley to recommend her removal to an asylum.^35^ Opposition from the Milners meant that she remained at The Knoll until the summer of 1884 when she went to stay with her aunt in Yorkshire. The family environment no longer offered a safe haven for her; within a month she had attempted to throw herself out of a window and so was committed to Barnwood House, a pioneering private mental asylum in Gloucestershire run by Dr Frederick Needham. She remained there for less than a year. Once again, she took a sudden dislike to her companion/maid and this seems to have encouraged fears that she was being ill-treated, a belief that was fostered by Needham’s policy of not permitting visitors to see patients unaccompanied. As a result, Needham asked for her to be removed from his care, insisting that the reputation of Barnwood ‘is of much more importance . . . than the presence or absence of any patient whatever her position or payment’. (HALS, DE/X317/F31, Frederick Needham to Miss Milner, 25 Mar. 1885).

## Ticehurst Hospital

Lady Durham was admitted to Ticehurst, a highly regarded private asylum in Sussex, in June 1885. As befitted an asylum specializing in treating the rich, living conditions at Ticehurst were luxurious. The buildings sat amidst extensive pleasure grounds that resembled the grounds of a stately home rather than a hospital. Amenities included a chapel, a bowling green, tennis courts, and aviaries. Carriages and carriage horses were available (at an additional cost) for the use of patients. Fees (and presumably the level of care) varied enormously. For the period covered by an extant register of patients (roughly covering the period 1895–1920) Lady Durham was the highest-paying patient. She (or rather Lord Durham on her behalf) paid £1,000 a year for her accommodation (two rooms) and in-house medical treatment. The use of a carriage, horses and a coachman cost an additional £150; consultations with external medical men, clothing, shoes and ‘fancy articles’ were charged extra (Wellcome, MS 6580).^36^ A description of the accommodation at Ticehurst made some 50 years earlier suggests that her rooms would have been comfortably furnished in the Victorian style – albeit with heavy perpendicular iron bars over the windows (Perceval, 1840: 90, 92). The case notes indicate that she was supervised by two attendants, one during the day and one at night; when her behaviour was very difficult, extra attendants were drafted in to assist them. Her actual treatment appears to have been minimal: drugs and enemas to relieve constipation, but otherwise she was simply encouraged (and probably forced) to go for daily walks and/or a drive in the carriage with an attendant, urged to play tennis and to listen to, or participate in, the asylum’s musical activities.^37^

Witnesses at the trial had referred to Lady Durham’s delusions while at the Knoll, but the nature of the delusions remained elusive (although this was not so when she was at Barnwood). When she was admitted to Ticehurst, Needham acted as one of the two doctors required to certify lunacy. He stated:

Rambling conversation. Numerous delusions, such as that she has committed great crimes, & is to be put to death. She refuses food, & has to be forcibly fed. Laughs in a childish manner and frequently refuses to speak or to answer questions. Is sometimes very violent.

Needham’s assistant, Henry Waddy, added that:

She is wandering & almost incoherent in conversation. She told me she had committed murder & had been placed in Private Prison for so doing; she could not remember who it was she killed, nor where it was that she committed the murder. She also said she had committed suicide.

Her initial examination at Ticehurst concluded that ‘she labours under mania – with which a considerable element of hysteria is mixed’ and that she had to be dressed and undressed. Her physical health was good, with the exception of ‘great constipation’ (Wellcome, MS 6329/537).

At the trial witnesses on both sides had concentrated on Lady Durham’s capacity for social interactions. The doctors at Ticehurst never remarked on her social skills or lack thereof, partly through lack of interest but also because she presented far more extreme and conspicuous symptoms. At her admission, ‘all evidences were obscured by intense obstinacy and resistance to every influence brought to bear. The patient maintained a dogged silence and threw herself into a rigid and resisting condition’. She showed no signs of excitement or exaltation, but depression was deduced from her worried look. As for her physical condition, it was noted that that her pulse fluctuated, her eyes were dilated and that she lay ‘in a constrained position, passively & actively resistant’, with her breathing irregular and constrained by hysterical spasms. Because of her refusal to eat she had been fed through a stomach pump (Wellcome, MS 6390, pp. 58–9).

During her first months at Ticehurst Lady Durham was closely monitored, with reports on her condition made on an almost daily basis. They provide considerable detail about her inability to cope with her new situation. Initially, she would not dress herself or change her clothes, and resisted any attempt by her attendants to dress or undress her; she also had to be carried from her bedroom to her sitting-room. Although she would go for walks, she frequently refused to return and had to be put into a chair or carriage and either carried or driven back and then carried to her rooms. Simply touching her wrist was enough to put her into ‘a rigid state’. On one occasion she put her hand through a glass door when the night attendant tried to prevent her from leaving her room at 1 a.m. She ‘will sit for hours doubled up with her head bent forward and will not speak a word when addressed . . . laughs in a meaningless manner and will stand staring in a vacant kind of manner without taking notice of anything’, or sometimes lay on the floor in a corner of the room ‘if allowed’. After a month of resistance and non-cooperation she went further and attacked her attendant. A week later she smashed several windows, and when she was prevented from picking fruit in the garden ‘she was most vindictive broke off the tops of some lilies, also threw stones to smash the hothouse roof and eventually walked a long way down the lanes and then sat down and refused to return’. On another occasion she described a nightmare in which she saw ghosts and her mother cutting her throat with a knife. Such nightmares seem to have been recurrent (Wellcome, MS 6390, pp. 59–62, 65, 72, 73).

Perhaps her behaviour reflected her difficulty in dealing with yet another change in her life, but it soon became apparent that her mental health problems were far more complex than had been revealed at the trial. Some of her delusions had been identified during her stay at Barnwood. In June 1886, a year after her admission to Ticehurst, Henry Maudsley was called in when one of her attendants reported that Lady Durham was experiencing auditory hallucinations, ‘listening to and answering persons in a picture on the wall in her bedroom’. Maudsley had seen her three months earlier and had been cautiously optimistic, but now he noted that he had suspected that she heard voices and warned that such hallucinations were likely to be permanent and could lead to ‘sudden, incalculable and sometimes violent acts’ (Wellcome, MS 6390, Henry Maudsley, 24 Mar., 21 June 1886, between pp. 255–6 and 257–8).

By the summer of 1887 Lady Durham was said to be much improved: she was ‘less insane in her behaviour’ so was able to drive and/or walk out daily ‘without any trouble’. Similarly optimistic reports were sent to her family (HALS, DE/X317/F9, Dr HF Hayes Newington to Mary Gerard, 1 Nov. 1885, 14 Mar., 16 May 1886, 6, 13, 27 Mar., 10 Apr., 15 May 1887). In reality, she was still subject to delusions and to auditory hallucinations that kept her awake at night. In autumn 1888 she accused her attendants of stealing her clothes and jewellery, using foul language and eating her menstrual discharge. Theodore Newington, the Resident Medical Officer, recorded that the allegations of theft were ‘the old delusions’ and that he had been present at the time of the alleged foul language and had heard nothing. She was openly masturbating, talking in ‘a very obscene manner’ and was convinced that men were gazing at her in the bath and followed her to the WC. She even insisted that Dr Hayes Newington, one of the co-owners of Ticehurst, was an impostor (Wellcome, MS 6391, pp. 171–82; MS 6392, pp. 303–306; MS 6393, pp. 119–30; MS 6397, pp. 251–3).

The last report of violence towards her attendants was not until May 1890. By that time the reports had become less and less frequent and somewhat formulaic; they described an unrelentingly inconsistent pattern of behaviour. Lady Durham sometimes spoke rationally, but her conversation was usually rambling and disconnected. An example of her incoherent thinking is provided by a letter to her sister in September 1895. It opens with a few coherent sentences before becoming a series of disconnected and bizarre remarks, including ‘Do you draw? No I cannot. What is all this? Anything like the heat I never felt. This child is seriously ill. Which child. Delirium tremens. You must have your head shaved it is good for the brain’.^38^

Her mood swings were sudden and unpredictable: periods of calm were punctuated by threats and actual acts of violence against both people and property. Usually, the people she attacked were hospital employees, but on at least one occasion she leaned out of her carriage to attack some children in a ‘most spiteful and vicious’ manner. On 31 January 1886 the notes recorded that she ‘has been talking a good deal about murdering people which is always a bad sign. Says “she thinks all useless persons ought to be killed to make room for the useful ones, and that everyone commits one murder during their life.”‘ It was indeed a bad sign; later that evening she smashed seven or eight panes of glass ‘in order, as she said to get more air’. She was also capable of inventing stories of the ‘terrible cruelties’ that had been practised against her – though her body had no marks of violence (Wellcome, MS 6390, pp. 62–82, 259). One of the last entries in the case notes described her as ‘absolutely dull and apathetic, does not occupy herself and does not speak though she will answer in monosyllables when spoken to’ (Wellcome, MS 6397, p. 251). Surviving Milner family correspondence suggests that towards the end of the 1890s Mary Gerard became convinced that her sister was being neglected.^39^ In 1898 she took Lady Durham on several trips away from the asylum, probably reconnoitring alternative care arrangements. Lady Durham was discharged from Ticehurst as ‘relieved’ in February 1899 (HALS, DE/X317/F20, Durham to Mary Gerard, 1 Nov. 1899, 25 Jan. 1899; Wellcome, MS 6397, pp. 251–3).

## After Ticehurst

The contrast between the symptoms described at the trial and those detailed in the case notes acts as a warning to those who try to reconstruct even a rough and tentative diagnosis from sources that were not created for that purpose and which focus on aspects of treatment, like the obsession with constipation, that are of little interest in modern psychiatry. One suspects that a practitioner treating her today would be trying to identify the factors that triggered her meltdowns, but her doctors made no attempt to do so, other than sometimes asking her why she had acted in such a way. Nor did they document how long her psychotic episodes lasted. If ASD were the central issue, one might posit that being confined at The Knoll or subsequently in an asylum would re-establish structure and routine in her daily life and thus help to restore some sort of equilibrium. Clearly it did not. The evidence of the case notes suggests that it took several years before there was a significant reduction in incidents of violence and that her capacity for rational thought deteriorated rather than improved. What none of the available sources can tell us is how Lady Durham herself regarded her life in the asylum or how she perceived her own mental health, apart from the rather sad remark made a few weeks after her admission to Ticehurst, on a day when she was otherwise giggling and making no sense: ‘Do you ever cure maniacs?’ (Wellcome, MS 6390, p. 64).

Perhaps even a modern psychiatrist would have difficulty in disentangling her symptoms and arriving at a satisfactory diagnosis. The evidence at the trial makes it easy to suggest ASD, while that of the case notes suggests psychosis of the sort most readily associated with schizophrenia or perhaps schizoaffective disorder. Were both sets of symptoms caused by a single underlying disorder? Did the traumatic breakdown created by her inability to cope with the increasing complexity of social demands after her marriage trigger the psychosis, or was it present earlier?

The case notes are full and informative but the observations are filtered through the assumptions and perceptions of her doctors, highlighting some areas, sidelining others, and at a distance of over a century it is almost impossible to identify those filters. When they stated that Lady Durham had been troubled all night by her voices, did they mean that she had slept badly because of her interaction with the voices or that she was worried by what the voices said? One almost wonders whether the voices offered her some sort of refuge from real life. Real life involved constant surveillance. During her short marriage she had carved out time and space for herself by taking long walks alone. At Ticehurst, walks – like all other activities – were taken under the supervision of her attendants who were probably more like warders than nurses. The use of force was commonplace, as was the use of purgatives and enemas – both of which Lady Durham resisted (see, for example, Wellcome, MS 6393, pp. 121–2). The records of Barnwood House for the relevant period do not survive, but the revelation that she had been fed through a stomach pump there is also indicative of force. That her incoherent letter to her sister survives amongst the Ticehurst case notes suggests that it was intercepted and that a system of censorship was in operation.

Ticehurst may have had luxurious accommodation, beautiful grounds and splendid facilities, but in the final reckoning it was still a place of confinement where the windows were barred and patients had neither privacy nor personal liberty. Just how Lady Durham fared after she left Ticehurst remains unknown. Her whereabouts can be traced on successive censuses but, other than the brief entry on the 1901 census that deemed her to be ‘feeble minded’, we have no information about the state of her mental health. It seems unlikely that it had improved or that she was able to lead anything approaching a normal life – her status as a ‘boarder’ in an asylum in 1911 suggests that she was what we would now describe as a voluntary patient.^40^ She died in October 1931, having outlived her husband by some 18 months.^41^

## Notes

1. *Pall Mall Gazette*, 25 Feb. 1885.

2. *Belfast News-Letter*, 16 Sept. 1884, 28 Feb. 1885.

3. *Dundee Courier*, 13 Mar. 1885.

4. This somewhat romantic tale of star-crossed lovers was not supported by the evidence presented to the court. While it is clear that Burghersh had been among Ethel Milner’s many admirers and that her family disapproved of him because they considered him to be poor, the testimony does not suggest that he had been a serious suitor, and after the verdict was delivered he took steps to deny publicly that he had ever proposed. Compared to Durham (worth over £70,000 a year) Burghersh was indeed poor, but he was heir to an estate worth some £11,000 a year at a time when a working man’s income was likely to have been between £30 and £50 a year; *Birmingham Daily Post*, 11 Mar. 1885; *Complete Peerage*: G.E.C. and Gibbs, 1916, p. 561; 28b, pp. 583–4.

5. *Times*, 11 Mar. 1885: 5, 9.

6. Birmingham Daily Post, 11 Mar. 1885.

7. *John Bull*, 14 Mar. 1885.

8. *Liverpool Mercury*, 11 Mar. 1885: 5.

9. The Marlborough Club had been founded in 1868 by the Prince of Wales, who continued to patronize it almost daily until his accession to the throne. It had a restricted membership, drawn from high society, and very high annual membership fees; see Sheppard, 1960.

10. For a translation of Asperger’s paper, see Frith, 1991.

11. The formal file held at the National Archives merely contains details of the verdict and the various steps in process; TNA, Court for Divorce and Matrimonial Causes: J 77/319/9540.

12. *Aberdeen Journal*, *Freeman’s Journal*, *Western Mail*, all 26 Feb. 1885; *The Penny Illustrated Paper*, 7 Mar. 1885.

13. I use the term quasi verbatim as, in most of the accounts, the questions of counsel have been redacted so that the answers seem to be a continuous statement; some newspapers concentrated on the evidence of upper-class witnesses and omitted or summarized the testimony of servants and shopkeepers.

14. For the speech, see *Times*, 16 Feb. 1883.

15. *Northern Echo*, *Bristol Mercury*, *The Standard*, all 28 Feb. 1885.

16. Some of her letters survive in the family archive (HALS, DE/X317/F21). Further letters were admitted as evidence in court. Referring to them in his judgment, Hannen concluded that they showed her to be ‘of low intellectual powers but . . . capable of receiving the ordinary education of young ladies of her class’; *Times*, 11 Mar. 1885.

17. *Times*, *Daily News* and *The Standard*, all 28 Feb., 2, 5 Mar. 1885.

18. Testimony of Lady Cecilia Webbe and George Nathaniel Curzon, *Times*, 6 Mar. 1885.

19. *Times*, 27 Feb., 5 Mar. 1885.

20. For Durham’s testimony, see: *Times*, *Daily News* and *The Standard*, all 26, 27 Feb. 1885.

21. The insinuation of attempted suicide was almost certainly relayed to the court by her doctors who gave evidence *in camera.* None of the newspaper reports included it until it was mentioned by Hannen in the course of his judgment; *Times*, 11 Mar. 1885.

22. *Belfast News-Letter*, 16 Sept. 1884;

23. *Times*, 11 Mar. 1885.

24. In his judgment, Hannen dismissed this remark as inconsequential, saying that ‘it is not unusual at the present day for young men and women to apply such terms as “dreadful” and “awful” without any consideration of their fitness’; *Times*, 11 Mar. 1885.

25. *Daily News*, 26 Feb. 1885; *Northern Echo*, 26, 27 Feb. 1885; *Manchester Times*, 28 Feb. 1885.

26. *Northern Echo*, 26 Feb. 1885; *Times*, *Daily News* and *The Standard*, 27, 28 Feb. 1885.

27. At her admission to Ticehurst in June 1885, she weighed 8 stone 4 lbs (Wellcome, MS 6390, p. 60). Difficulty in getting her to eat and consequent constipation are recurrent themes of the medical notes. See also HALS, DE/X317/F9, Dr HF Hayes Newington to Mary Gerard, 15 May 1887.

28. *Daily News*, 2 Mar. 1885; *York Herald*, 26 Feb. 1885.

29. *Daily News*, 26 Feb. 1885; *Northern Echo*, 26 and 27 Feb. 1885; *Manchester Times*, 28 Feb. 1885.

30. Mentioned by Hannen in the course of his judgment; *Times*, 11 Mar. 1885.

31. The later case notes also mention her habit of knitting ‘which she pulls to pieces and works over again’; *Daily Gazette for Middlesbrough*, 26, 28 Feb. 1885; *Times*, 26 Feb. 1885; *Daily News*, 28 Feb. 1885; *Berrows Worcester Journal*, 7 Mar. 1885; Wellcome, MS 6392, p. 304.

32. *Daily News*, 5 Mar. 1885; *Northern Echo*, 5 March 1885.

33. As noted below, she took a similar aversion to her companion/nurse at Barnwood. Sudden, and sometimes temporary, dislikes of her companions, maids and attendants, accompanied by threats and actual violence, are a recurrent theme of her medical notes whilst at Ticehurst Hospital and were also mentioned by Dr Hayes Newington, one of the co-proprietors of Ticehurst, in his letters to Mary Gerard. There is no indication in any of the sources that she was ever violent to her own family; HALS, DE/X317/F9, Dr HF Hayes Newington to Mary Gerard, 6 May 1886.

34. *Daily News*, 9 Mar. 1885

35. *Times*, 26 and 27 Feb. and 5 Mar. 1885.

36. For a detailed breakdown of fees see, for example, Wellcome, MS 6567, pp. 461–4. Repeated references to her breaking glass panes in doors, as well as windows, suggest that the two rooms were separated by a glass-paned door, enabling constant surveillance, even after it had been deemed no longer necessary for an attendant to spend the night actually in her bedroom.

37. A volume of notes on members of staff suggests that an interest in sport or the ability to play a musical instrument enhanced an individual’s chances of employment; Wellcome, MS 6523.

38. Wellcome MS 6393, Ethel Milner to Mary Gerard, 28 Sept. [1895], inserted between pp.127–8. She had been in the habit of signing her letters as Ethel E L Durham but this one is signed Ethel E L Milner.

39. The case notes indicate that she was no longer being personally supervised by Dr Hayes Newington. It is also likely that at this time Hayes Newington was increasingly distracted by problems concerning the future management of Ticehurst, which was essentially a family business and subject to an unwieldy trust. That the subject of the future disposition of the business was already an issue in the later 1890s is evident from his wife’s letters to Mary Gerard; see MacKenzie, 1992: 163–7; HALS, DE/X317/F9, Mrs J Hayes Newington to Mary Gerard, 18 and 22 Jan [1899].

40. The initial arrangements for her to live in Herne Bay fell apart. In 1901 she was living in a hotel in Tunbridge Wells with a maid and two companions (one of whom had been the superintendent of Highlands, the house in which Lady Durham had lived at Ticehurst). Lady Durham was described on the 1901 census as ‘feeble minded’ (on the 1891 census when she was in Ticehurst Hospital she had been described as lunatic). In 1911 she was a boarder rather than a patient in Camberwell House in Peckham. Camberwell House was a large and well regarded asylum set, like Ticehurst, in extensive grounds; TNA: RG 12/778,Ticehurst Hospital; RG13/752, The Mount Ephraim, Earls Court; RG14/2619, Camberwell House; Wellcome, MS 6393 p. 128; Blanch, 1877: 348–9.

41. *Times*, 31 Oct. 1931.
